# Assessing the health impact of transnational corporations: its importance and a framework

**DOI:** 10.1186/s12992-016-0164-x

**Published:** 2016-06-15

**Authors:** Frances E. Baum, David M. Sanders, Matt Fisher, Julia Anaf, Nicholas Freudenberg, Sharon Friel, Ronald Labonté, Leslie London, Carlos Monteiro, Alex Scott-Samuel, Amit Sen

**Affiliations:** Southgate Institute of Health, Society and Equity, Flinders University, GPO Box 2001, Adelaide, SA 5001 Australia; School of Public Health, University of the Western Cape, Bellville, 7535 South Africa; School of Public Health, City University of New York, New York, NY 10033 USA; Regulatory Institutions Network, The Australian National University, Canberra, ACT 2601 Australia; School of Epidemiology, Public Health, and Preventive Medicine, University of Ottawa, Ottawa, Ontario KIH 8M8, Canada; School of Public Health and Family Medicine, University of Cape Town, Cape Town, Observatory, 7925, South Africa; Center for Epidemiological Research in Nutrition and Health, University of São Paulo, Sao Paulo, SP Brazil; Department of Public Health and Policy, University of Liverpool, Liverpool, L69 3GB, UK; People’s Health Movement, New Delhi, 110017 India

**Keywords:** Health impact assessment, Health inequalities, Public health policy, Health promotion, Methodology

## Abstract

**Background:**

The adverse health and equity impacts of transnational corporations’ (TNCs) practices have become central public health concerns as TNCs increasingly dominate global trade and investment and shape national economies. Despite this, methodologies have been lacking with which to study the health equity impacts of individual corporations and thus to inform actions to mitigate or reverse negative and increase positive impacts.

**Methods:**

This paper reports on a framework designed to conduct corporate health impact assessment (CHIA), developed at a meeting held at the Rockefeller Foundation Bellagio Center in May 2015.

**Results:**

On the basis of the deliberations at the meeting it was recommended that the CHIA should be based on ex post assessment and follow the standard HIA steps of screening, scoping, identification, assessment, decision-making and recommendations. A framework to conduct the CHIA was developed and designed to be applied to a TNC’s practices internationally, and within countries to enable comparison of practices and health impacts in different settings. The meeting participants proposed that impacts should be assessed according to the TNC’s global and national operating context; its organisational structure, political and business practices (including the type, distribution and marketing of its products); and workforce and working conditions, social factors, the environment, consumption patterns, and economic conditions within countries.

**Conclusion:**

We anticipate that the results of the CHIA will be used by civil society for capacity building and advocacy purposes, by governments to inform regulatory decision-making, and by TNCs to lessen their negative health impacts on health and fulfil commitments made to corporate social responsibility.

## Background

A major challenge for public health in the twenty-first century is to respond to the changing dynamics of capitalist economies and the attendant impacts on people’s daily living conditions, and ultimately health equity. Central to this process has been the growth in the power and influence of transnational corporations (TNCs). Since TNCs increasingly dominate global trade and investment and shape national economies, the adverse health and equity impacts of their practices are now fundamental influences on public health. Despite this, researchers have not yet developed the tools or methods with which to study the health equity impacts (both positive and negative) of individual corporations and thus to inform actions to mitigate and reverse negative health impacts and reinforce any positive impacts. This paper reports on the process of developing a framework designed to conduct corporate health impact assessment, the insights generated by this process and the resultant framework, developed at a meeting held at the Rockefeller Foundation Bellagio Center in May 2015. The paper also discusses ideas canvassed at the meeting regarding the feasibility of conducting such impact assessment and its likely benefits to public health. The workshop included experts on TNC practices, the health impact of TNCs in the food and mining sectors, the evaluation and assessment of health impacts, and civil society activists who advocate reversing adverse impacts.

### Public health significance of TNCs

**Fig. 1:**
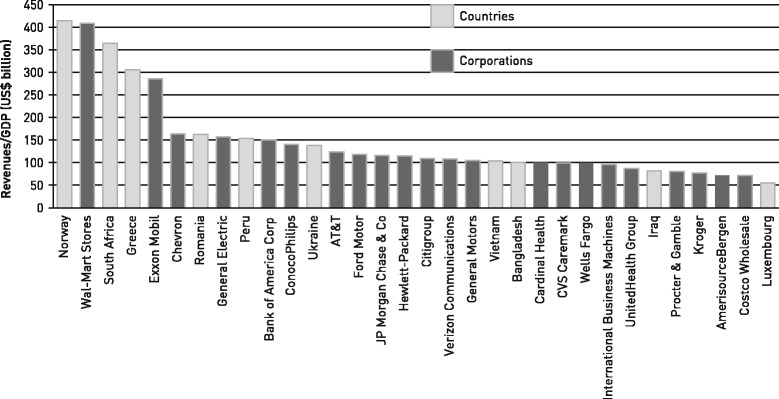
Comparing the size of the world’s largest corporations with selected countries.^2^

Transnational corporations (TNCs) are incorporated or unincorporated enterprises operating across multiple countries comprising parent enterprises and their foreign affiliates. It is estimated that there are now over 100,000 TNCs operating globally [1]. Many TNCs are economies which are larger than those of national states (see Fig. 1) [2]. Of the 100 governments and corporations with the highest annual revenues in 2014, 63 are corporations [3] and 37 are governments [4]. This growth is facilitated by the broader global context which promotes neoliberal policies, including trade liberalisation, producer subsidies and strengthened private property rights. The growth is also driven by growing demand for TNC products in developing countries. A growing body of research has examined the practices of TNCs in sectors such as food and beverage, tobacco, pharmaceuticals and extractive industries. It shows that TNC products and operations can support gains in public health through investment in host countries which contributes to improvements in employment opportunity, working conditions, education, infrastructure or health service provision [5]. Some TNCs also undertake practices intended to assess and improve performance in areas of environmental impact, social accountability or ‘shared value’ [6]. Freudenberg argues, however, that over the past two decades world economic arrangements have been increasingly changed to suit the needs of corporations and “as a result set the stage for the twenty-first century disease epidemics” [7]. The UN Special Rapporteur on the Right to Food said that the global food system which is largely run by TNCs “is a public health disaster” [8]. The Commission on the Social Determinants of Health [9] noted that binding trade agreements together with increasing corporate power and capital mobility have diminished individual countries’ capacities to ensure that economic activity contributes to health equity, or at least does not undermine it. The Lancet—University of Oslo Commission on Global Governance for Health noted that “Private firms have an influential role in contemporary global governance. Large transnational companies wield tremendous economic power, which they can deploy to further their interests in global governance processes and global markets” [10]. The Commission went on to note that, although there were benefits from the operation of TNCs, “they can also harm health through dangerous working conditions, inadequate pay, environmental pollution, or by producing goods that are a threat to health (e.g., tobacco)” [9]. In addition to these powerful voices the Director General of the World Health Organization [11] has pointed to the power of TNCs and the ways in which they can influence the public health agenda adversely. An increasing amount of research indicates that while there are some positive effects there are significant negative impacts on health from corporate structures, products and practices (see Table 1).

**Table 1:** Examples of TNC impacts on health

Transnational corporations operate in many sectors including food and beverages, extractive industries, tobacco, alcohol and pharmaceuticals. They have the capacity to both promote and harm health. Examples of beneficial health impacts from TNCs include a range of shared value initiatives: • Mars (chocolate) engaging in sustainable cocoa initiatives through employing science, technology, and certification to assist farmers through increasing yields and sustainable supply [21]. • Nestlé adding micronutrients including iron and iodine to foods to improve health in impoverished regions [22]. • BHP Billiton improving the quality and reliability of local suppliers through the “World Class Supplier Program” in Chile, leading to significant employment growth [23]. Examples of adverse health impacts from TNCs are: • In 1998, at a time when the largest number of HIV/AIDS afflicted people lived in South Africa, 41 transnational drug companies sued the government of South Africa for initiating measures to reduce prices of anti-retrovirals [24]. • Coca Cola’s depletion and pollution of groundwater in India to make a product with 10 teaspoons of sugar per serving, contributing to global epidemics of obesity and diabetes [25]. • In June 2009 an outbreak of E.coli food poisoning in the United States was linked to Toll House refrigerated cookie dough produced by Nestlé at a plant in Danville, Virginia. The company recalled all Toll House products in the country, but it came to light that the plant had previously refused to give inspectors from the federal Food and Drug Administration (FDA) access to internal records relating to matters such as pest control and consumer complaints [26]. • The 1984 toxic gas leak from the Union Carbide chemical plant in India, which included the loss of life of thousands of people in Bhopal where the community still suffers the aftermath and is campaigning for adequate clean-up, compensation and justice [27]. • Philip Morris Asia Limited sued the Australian government to repeal plain packaging laws despite the fact that 1 billion tobacco-related deaths are predicted globally this century [28]. • Tax avoidance strategies by McDonald’s global operations have potentially cost European governments 1.0 billion Euros and the Australian Government $497 million dollars in unrealised receipts between 2009 and 2013 alone, reducing amounts government have to invest in health promoting infrastructure and services [29]. • Extractive industries have huge negative environmental and social impacts. Since the Australian TNC BHP began mining in Papua New Guinea in the 1980s, hundreds of millions of tons of waste have been dumped into the Tedi River causing irreversible damage to the river ecology and mass deforestation of surrounding areas and resultant health impact on Indigenous peoples [30]. • A narrative review indicated that pharmaceutical corporations suppress and misinterpret scientific evidence which leads to systematic overestimation of the safety and efficacy of products, and also exerts pressure on regulatory bodies against disclosure of adverse effects which are deemed to be ‘trade secrets’ [31].
These examples are indicative, but not exhaustive, of the scope of cumulative local, regional, national and global health impacts that potentially result from the activities of TNCs. They are also indicative of the ways in which the economic power of TNCs is likely to influence the pressures on governments and other stakeholders to make trade-offs between economic and social goals within processes of national development.

Despite such studies there has been no research that has developed an overarching analytical framework and systematic methodology that can be used to assess the impacts of TNC practices on populations within countries from a health equity perspective.

## Methods

### Bellagio meeting: developing a corporate health impact assessment

Our meeting was held in Bellagio in April 2015 and was attended by representatives from academia, civil society and TNCs. Its main aim was to discuss the usefulness and feasibility of conducting a corporate health impact assessment with a focus on its application in low and middle income countries. The meeting oriented its discussion on the practices and health impacts of corporations in the food and beverage (excluding alcohol) and extractive industry sectors. Discussions were held on the viability of developing and implementing a corporate health impact assessment from the perspectives of health equity researchers; of activists campaigning against negative health aspects of corporate practices and corporations globally, nationally or within particular communities; and corporations representative of our two focal sectors (food/beverage and extractive industries). This follows development of a range of other tools being used to assess TNC performance [6, 12, 13]. Our CHIA framework augments these other approaches because it seeks to assess individual TNCs across the full scope of their structures and practices; provides a tool to compare a TNC’s practices across countries; and is focused specifically on health and health equity impacts. Workshop participants agreed that corporations have both positive and negative impacts on health, and that government oversight is important in minimising the negative impacts. Civil society activists and researchers stressed significant detrimental environmental impacts, dislocation of traditional communities, unsafe working conditions leading to high rates of injury and death, and the adverse impact of unhealthy products. The corporate sector representatives emphasised the potential for gains in areas such as employment standards, workplace safety and environmental sustainability, and through corporate policies to address social responsibility or create ‘shared value’.

Although there was some concern about the processes that would be involved in a corporate HIA all participants agreed that such a tool would be helpful especially as it would focus on the operations of individual corporations including their entire supply chain. Table 2 describes the ways in which this was seen to be the case by different groups of participants.

**Table 2:** Predicted value of Corporate Health Impact Assessment

Predicted value to researchers
Evidence to inform public policy decisions
Evidence elucidating the health and health equity impacts of individual TNCs’ structures, products and practices; and how these differ between countries
Understanding of how TNC practices affecting health are influenced by international and national regulatory structures
Predicted value to civil society activists
Advocacy tool to enhance community capacity to understand and engage on issues associated with health impact of TNC operations
Facilitate community involvement in debates about TNC health impact and possible government response
Predicted value to corporations
Evidence to inform corporate policies and practices to reduce adverse and optimise positive impacts on health and health equity within their countries of operation; and to achieve greater equity of practices across countries
Predicted value to governments and policy-makers
Evidence to inform policy decisions regarding project approval and appropriate regulation

Participants also discussed the criteria by which corporations would be selected for corporate health impact assessment (CHIA). This decision would be informed by a consideration of the characteristics of the industry sectors in which a TNC operates, and the characteristics of the TNC itself (including brand profile, the countries in which it operates and its scope of operations across a supply chain). Further considerations are the type and likely quality of information on the TNC’s operations, the capacity of the research team within the countries in which the TNC operates, and the level of civil society interest. Given our interest in the impact of regulation it would also be desirable to select TNCs which operate in countries with widely varying regulatory environments.

Considerable discussion was held about how effective engagement with affected communities within countries could be integral to the conduct of a CHIA. Recognising that HIA methodology has been criticised for being very technical and, for example, not treating community knowledge as legitimate, the meeting was adamant that effective CHIA would rely on at least effective community engagement processes and in some instances should be community-driven when the capacity exists within civil society.

### Assessing health impact

Health impact assessment (HIA) has been used over many years to produce evidence-based recommendations to inform decision-making about proposed projects, plans, programs and policies in order to maximise their positive and minimise their negative impacts on health. HIA stresses the importance of defining health impacts broadly by including social, environmental and economic factors that determine health and health equity outcomes [14–17]. The workshop was interested in the adaptation of HIA to consider health impacts either concurrently or retrospectively (*ex post*) in terms of existing or past TNC practices, or prospectively and predictively (*ex ante*) to ascertain likely impacts when new operations are proposed. An *ex ante* HIA forecasts potential impacts as part of the planning, design and approval of an intervention. *Ex post* assessment identifies actual impacts during and after implementation [18, 19] and provides an evidence base for corrective actions to be taken if necessary, and to inform *ex ante* HIAs. HIA incorporates five steps: screening, scoping, identification, assessment, decision-making and recommendations, as well as evaluation and follow-up. In examining the feasibility of applying HIA to corporate practices the workshop acted as the scoping stage of a HIA process. It also then developed a framework within which the identification and assessment stages could be conducted.

## Results and discussion

### Framework for corporate health impact assessment

**Fig. 2:**
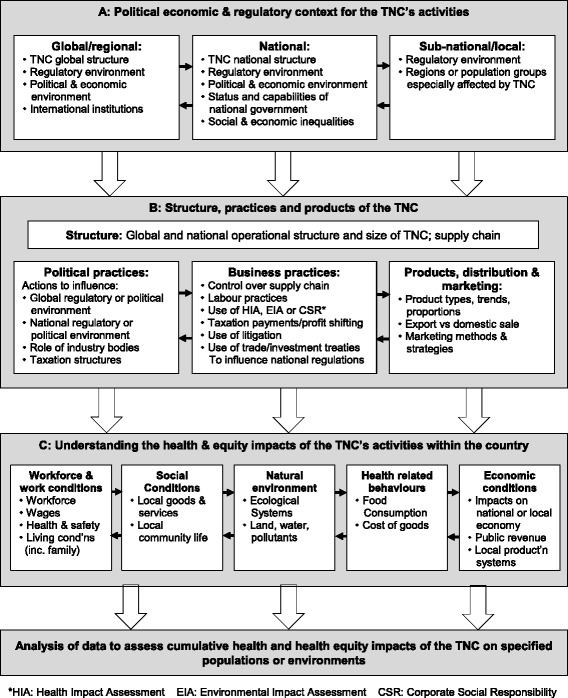
Framework for conducting a corporate health impact assessment

A draft framework to guide the design and implementation of CHIA was presented to the participants on the first day of the meeting. This was then extensively revised over the following two days, through detailed discussion about the importance of understanding the direct and indirect mechanisms by which TNCs affect health. The discussion also highlighted the importance of studying a TNC’s global, national and local operations and so emphasising the need for the framework to be adaptable to different contexts. This discussion resulted in the framework shown in Fig. 2.

An overview of the content of the framework is provided below:

A. **Context**: global operating context (history of TNC; effect on TNC of global regulatory environment including trade agreements; mapping of links between political and TNC elites); supply chain analysis spanning primary production, processing, manufacturing, transport, retail and consumption; national operating environment (agreements between TNC and government, description of national regulatory environments, national corruption index, activities of industry associations); regional community or groups affected by the TNC’s activities within the LMIC (description of regions and communities and groups affected).
B. **Structure**, **products and practices of TNCs**: organisational structure, political financing and lobbying practices, and business practices including board membership and business affiliations, corporate philanthropy and sponsorships, use of health impact assessment (HIA), environmental impact assessment (EIA) and TNC products including energy and nutrient components, and distribution and forms of creative and integrated marketing especially targeted to children and young people.
C. **Health impact of the TNC within the country**: These categories allow for the possibility of recognising both positive and adverse impacts on health in any of these domains:
  ***Workforce and working conditions*** (eg. description, occupational health systems, remuneration of workers in relation to cost of living indexes, extent of unionisation, quality of provision of health care and impact on social determinants of health such as housing).
  ***Social conditions*** (eg. impact of TNC goods on locally produced goods and services and net employment levels, impact of operation on local living conditions, the value of corporate social responsibility initiatives, social dynamics created by TNC operations including impact of fly-in-fly-out workers, impacts on social, cultural and spiritual life, and the impact of migrant labour in mines affecting sexual practices).
  ***Environment*** (eg. impact on natural systems in ways that affect health or health risk, including air/water quality, exposure to pollutants, land clearing, energy consumption, water, waste disposal).
  ***Consumption patterns*** (eg. impact of quality and consumption of TNC goods on health, national marketing practices).
  ***Economic mediated impact on health*** (eg. impact on TNC operations on overall economic conditions including tax revenues, reliance and vulnerability of national economy on TNC, economic and health impacts on local businesses/farmers).

Standard HIA data-gathering methods include literature sources, and collection of quantitative and qualitative information, including both primary and secondary data. Primary data are used explicitly for the HIA, such as community consultations or stakeholder interviews. Secondary data include relevant peer reviewed articles and routine data collected by companies or governments that is used for other purposes. Following standard data gathering methods, evidence relating to each domain in the framework would be collected through a mix of: analysis of key documents (such as corporate policies, reports and media releases, government or international agency reports); interviews with key players (including corporate actors, lobbyists, current and former political staff, members of non-government organisations and affected communities within the countries concerned); and searches of databases for evidence on impacts of the TNCs on health. The information collected in each of the above domains would be analysed to determine the likely impacts of the TNC’s operations on overall population health and health equity. This analysis would also determine which groups within the affected country would be particularly subject to adverse health impacts and which would gain health benefits from the TNC’s operation.

The framework is designed to be applied to corporations within countries. We anticipate that the CHIA will have to cover very different contexts and will have to be adapted to local conditions. We acknowledge that in some countries it will be easier to collect a robust set of data than in others. Applying the CHIA framework to the operations of one TNC within several countries would then enable a comparison of health impacts in different national settings, and some assessment of the overall impact of the TNC. A major advantage of the cross-national comparison was seen to be the ability to determine from this information the impact of different national regulatory structures on the ways in which corporations operate. It would also permit comparisons among different corporations.

The meeting considered the CHIA framework to be most likely used by civil society activists, with academic research support, for advocacy purposes. However it was noted at the meeting that TNCs are increasingly concerned about their public image in response to concerns expressed by activist civil society groups, shareholder activism and potential employees who desire to work for an ethical company. Consequently, in some circumstances TNCs may choose to co-operate with the CHIA process (e.g. sharing more of their internal documents and participating in the assessment itself) and to use the results to inform their own operations. In the event TNCs decide not to co-operate the CHIA process would still be possible from publicly available documents, media and data collection from those experiencing the health impacts. As local and national governments develop capacity in HIA [20], some may choose to apply this approach to corporate practices as well as to public projects. The necessary skills and capacities for undertaking a CHIA include a multi-disciplinary team, legal expertise on the structure and operations of TNCs, understanding the role and practice of health impact assessments, and public health expertise to determine the health impacts. Other necessary expertise includes that relating to the products or practices of particular types of TNCs; for example nutrition experts for food industry TNCs and occupational health and safety expertise for extractive industry TNCs. A civil society perspective is also necessary for informing a CHIA.

## Conclusions

In our continually globalising world, TNCs have a powerful impact on health and well-being in almost every country on earth. This impact is mediated through the business and political practices of the TNCs, the types of regulatory environments in which they operate and over which they have increasing influence, TNCs’ employment and environmental practices, and the ways in which their operations affect communities, regions and countries. There has been very little systematic investigation of the health equity impacts of these corporations. Our Bellagio meeting enabled a detailed consideration of the value, challenges and practicalities of conducting such systematic studies using the methodology we propose. It suggested that there will be significant benefits in documenting these health effects through a formal corporate health impact assessment process. The meeting was clear that the CHIA should be linked to the overarching aim of reducing economic inequality and social injustice. The results would be available for use by civil society advocates, corporations who wish to lessen the adverse health impact of their operations and by governments who would be able to assess different regulatory frameworks according to their ability to reduce adverse health and equity impacts and/or enhance health benefits of TNC operations.
